# Understanding the role of scientific evidence in consumer evaluation of natural health products for osteoarthritis an application of the means end chain approach

**DOI:** 10.1186/1472-6882-12-198

**Published:** 2012-10-30

**Authors:** Teresa Tsui, Heather Boon, Andreas Boecker, Natasha Kachan, Murray Krahn

**Affiliations:** 1 Leslie Dan Faculty of Pharmacy, University of Toronto, Toronto, ON, Canada; 2 Department of Food, Agricultural and Resource Economics, University of Guelph, Guelph, ON, Canada

**Keywords:** Natural health products, Decision-making, Means ends chain analysis, Osteoarthritis

## Abstract

**Background:**

Over 30% of individuals use natural health products (NHPs) for osteoarthritis-related pain. The Deficit Model for the Public Understanding of Science suggests that if individuals are given more information (especially about scientific evidence) they will make better health-related decisions. In contrast, the Contextual Model argues that scientific evidence is one of many factors that explain how consumers make health-related decisions. The primary objective was to investigate how the level of scientific evidence supporting the efficacy of NHPs impacts consumer decision-making in the self-selection of NHPs by individuals with osteoarthritis.

**Methods:**

The means-end chain approach to product evaluation was used to compare laddering interviews with two groups of community-dwelling Canadian seniors who had used NHPs to treat their osteoarthritis. Group 1 (n=13) had used only NHPs (glucosamine and/or chondroitin) with “high” scientific evidence of efficacy. Group 2 (n=12) had used NHPs (methylsulfonylmethane (MSM) and/or bromelain) with little or no scientific evidence supporting efficacy. Content analysis and generation of hierarchical value maps facilitated the identification of similarities and differences between the two groups.

**Results:**

The dominant decision-making chains for participants in the two scientific evidence categories were similar. Scientific evidence was an important decision-making factor but not as important as the advice from health care providers, friends and family. Most participants learned about scientific evidence via indirect sources from health care providers and the media.

**Conclusions:**

The Contextual Model of the public understanding of science helps to explain why our participants believed scientific evidence is not the most important factor in their decision to use NHPs to help manage their osteoarthritis.

## Background

Natural health products (NHPs) are defined in Canada as products made from ingredients found in nature sold over-the-counter for medical purposes [1] including herbal medicines, vitamins and minerals. It is an estimated 3 billion dollar industry annually in North America [2]. Just over 70% of Canadians reported *ever* using a NHP as of 2005 and 38% reported the daily use of NHPs [3]. Similar statistics are reported for complementary and alternative medicine (CAM [1]) use throughout the Western world – with approximately 40% of individuals currently using CAM, and 70% reporting past use in Europe, Australia, and the United States [4,5].

Osteoarthritis (OA) is the most common form of arthritis, affecting about 18% of females and 10% of males over the age of 60 years worldwide. OA is a condition responsible for significant chronic disability as a result of joint degeneration and reduction in mobility. Approximately 60% of seniors with OA report using non-prescription medications to treat their OA [6] and 30-45% report using NHPs [6,7]. The widespread self-management of OA leads one to question how consumers choose which products to purchase.

There are conflicting studies regarding the role scientific evidence plays in influencing consumer choices [8,9]. Recommendations of trusted individuals such as health care providers (e.g., pharmacist, doctors), and friends and family appear to play an important role in non-prescription decision-making [10]. Yet previous studies of consumer decision-making related to CAM have suggested that individuals review and prioritize scientific evidence as a decision-making factor [11-13]. Additionally, lack of scientific evidence or evidence of ineffectiveness has been reported to sway consumers away from using CAM therapies [14,15]. Thus it remains unclear how scientific evidence fits into consumers’ decisions about NHPs.

Much consumer education focuses on giving people “the evidence” about the safety and efficacy of various treatment modalities, assuming that armed with this information they will make the “right choice”. This view is consistent with the Deficit Model for the Public Understanding of Science [16]. Underlying this model is the assumption that the public trusts scientific experts, institutions, and perceives that scientific information is useful and relevant to their decision-making process [16]. Another assumption is that the assimilation of scientific knowledge into the decision-making process is directly correlated to the amount of scientific knowledge an individual possesses [17,18]. The Deficit Model has been criticized for not providing adequate explanations for those individuals who have the scientific information and still choose not to use it in their decision-making process. According to the Deficit Model, the public’s doubts or concerns about scientific progress or innovation are due to misunderstanding or ignorance about the science that underlies it. While the Deficit Model as described here is somewhat simplified to enhance the clarity of this argument, it has been shown to underlie many of the public education campaigns related to health-related technology and treatments [17].

The Contextual Model was developed as a critique of the Deficit Model in an attempt to explain why some individuals make “unscientific” choices despite having scientific knowledge. McKechnie, a proponent of the Contextual Model writes: “scientific expertise is identified, experienced, and responded to in terms of the institutional, cultural, and social dimensions in which it is always inevitably embedded” [16]. Contrary to the Deficit Model, the Contextual Model cautions that trust in authority should not be assumed [19]. Therefore, even if “scientific” information is being conveyed by experts, the public needs to first be convinced that understanding scientific information is important [20], or else attempts to educate the public about the science are counter-productive.

The purpose of this paper is to explore how consumers use scientific evidence when choosing NHPs to manage their OA. The findings are discussed in the context of the Deficit and Contextual Models for the Public Understanding of Science.

## Methods

We used the means-end chain (MEC) approach to understand osteoarthritis-related NHP self-medication decisions [21-23]. The MEC approach, primarily used in marketing research, is designed to help consumers re-construct and verbalize what may have become largely routine product evaluation processes [24,25]. Its associated laddering interviewing technique enables eliciting how consumers link product attributes with their values and beliefs via the perceived consequences of the attributes to obtain cognitive maps of production evaluation and choice processes [22]. This project received approval from the Office of Research Ethics at the University of Toronto.

### Participants

Stratified convenience sampling and snow-ball sampling strategies were used to recruit 25 participants through seniors activity centres, community organizations, newspapers, magazines and an online community (craigslist). English speaking adults with a self-reported diagnosis of osteoarthritis of any joint for at least the last three months who had self-medicated with at least one NHP from the following list: glucosamine, chondroitin, MSM or bromelain in the last year, were invited to participate. These four products were selected because of their relatively common use and ease of access in the greater Toronto area, as well as to allow comparison of products with a relatively high level of scientific evidence of efficacy and products with a relatively low level of scientific evidence of efficacy. The aim of recruitment was to have two groups: one composed of participants who reported only using NHPs with high levels of scientific evidence (levels A or B as defined by the Natural Standard database [26]; in our case glucosamine and chondroitin) and one composed of individuals only using NHPs with little or no scientific evidence (level C or lower as defined by Natural Standard [26]; in our case bromelain and MSM). However, it was not possible to recruit participants who had never tried glucosamine or chondroitin due to the popularity of these NHPs in North America. Thus if a participant reported using any product lacking levels A or B evidence support (e.g., bromelain and MSM which have level C or lower evidence support), they were assigned to the “low” scientific evidence category. Individuals with previous professional health care training, employment with an NHP manufacturer or market research company, patients of any of the study investigators or over the age of 80 were excluded. The upper limit of age 80 was set based on our team’s previous experience with the laddering interview which can last up to 2 hours and be quite mentally and physically challenging, particularly for more elderly individuals. The sample was selected to include both men and women with a range of ages, levels of education and income. Written informed consent was obtained from all participants prior to each interview.

### Data collection

Face-to-face semi-structured interviews using the laddering process and lasting 60 to 90 minutes were conducted. Interviews were audio-recorded and transcribed verbatim. There were four general steps to the interview: 1) general questions inquiring about how participants found out about their NHPs; 2) the laddering interview, as described in detail below; 3) a brief semi-structured interview about participants’ views on scientific evidence; and, 4) socio-economic and demographic information collected via a standardized questionnaire. Each participant received a $50 gift card for participating in the study.

In the laddering section of the interview, participants were first asked to state what they like and dislike about the products which they *have used* in the past six months. Next, participants were asked about the remaining products they had heard of, but *not used* to find out why they did not use them. Based on these responses, the interviewer compiled a list of all specific product attributes salient to that individual participant.

Each product attribute formed the starting point of a ladder, linking the product attribute with its associated consequence(s) and the underlying personal value(s). Variations of the probing question “Why is that important to you?” were used to encourage participants to think more deeply about the meaning of each step, leading to increasingly abstract concepts being elicited [22,24,25]. A ladder was concluded when the respondent had reached the most abstract level of terminal values or was not able to answer the probing question.

### Data analysis

**Figure 1:**
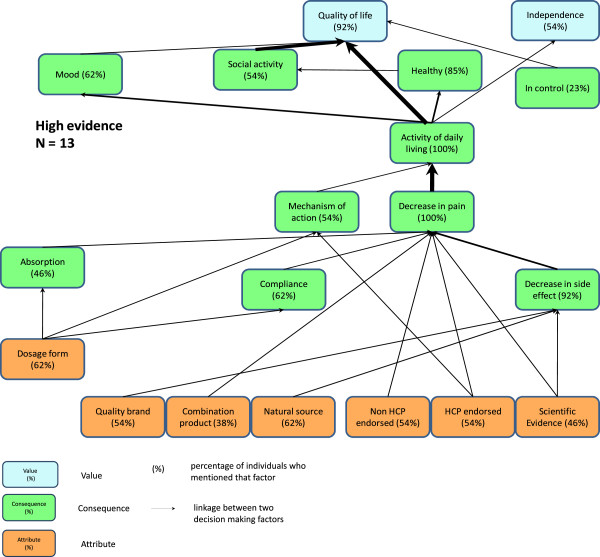
High Scientific Evidence Group Hierarchical Value Map.

**Figure 2:**
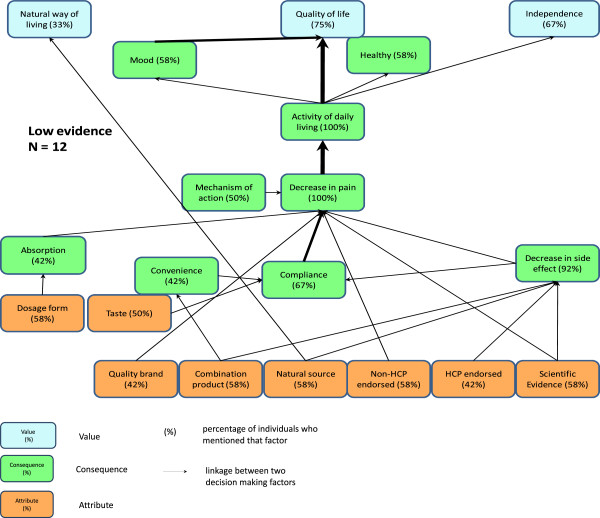
Low Scientific Evidence Hierarchical Value Map.

Analysis of laddering data proceeded in three steps. First, three coders independently identified attributes, consequences and values and the links between them in each respondent’s transcript. Second, for each participant the coded data were entered as ladders into the MECanalyst Plus [24] software, which summarizes the individual participant data in the so-called implication matrix that documents the number of links between all attributes, consequences and values that were identified in the first step. Third, the software was run to generate a graphic representation of the data referred to as hierarchical value maps (HVM) (See Figures 1 and 2). HVMs illustrate the links between individual concepts. The strength of a link is measured by the number of respondents that made that link in a laddering interview. The fundamental assumption of the MEC approach is that the strength of a link indicates how readily a more abstract concept (e.g., a value) is activated by a perceived consequence or attribute [22]. Links mentioned by very few participants are assumed to either represent idiosyncratic participant responses or play a minor role in product evaluation and decision-making [22]. A cut off defines a minimum number of mentions that a link must have to appear in the HVM. Increasing the cut off reduces the information content of the HVM – it is 100% at cut off one – but also enhances its tractability. The chosen cut off for the high and low scientific evidence HVMs was three which retained an acceptable 54% and 62% of the links, respectively.

Constant comparative content analysis was used to code discussions of scientific evidence and other sources of information throughout the interviews. Coding was completed independently by three investigators, with regular meetings scheduled every two to three interviews in order to ensure consensus between investigators. NVivo software was used to facilitate the content analysis [27].

## Results

### Overview of participants’ demographic characteristics

Of the 25 participants, 13 had only used NHPs in the high [2] scientific evidence category and 12 had used NHPs in the low [3] scientific evidence category. Overall, no statistically significant differences were found between participants in the high and low scientific evidence categories with respect to gender, age, ethnicity, education level, net household income, presence of extended health insurance, duration of using NHPs, perceived importance of scientific evidence, overall perceptions of health, or pain severity (See Table 1).

**Table 1:** Participant demographics and other characteristics

**Item**	**Everyone (N = 25)**	**High (N = 13)**	**Low (N = 12)**	**p-value**
**Gender**	19 (76)	10 (77)	9 (75)	1^**a**^
**Female, N (%)**				
**Mean Age in years (range)**	58.8 (42–78)	55.08 (42–78)	62.92 (43–78)	0.100 ^**b**^
**Ethnicity, N (%)**				0.422^**b**^
**North American**	10 (40)	6 (46)	4 (40)	
**European**	13 (52)	5 (38)	8 (62)	
**Other**	6 (24)	4 (67)	2 (33)	
**At least some college as highest education, N (%)**	15 (60)	9 (69)	6 (50)	0.428^**a**^
**Annual household net income less than $50,000**^**d**^**N (%)**	14 (56)	7 (54)	7 (58)	1^**a**^
**Have Extended health insurance**, **N (%)**	12 (48)	6 (46)	6 (50)	1^**a**^
**Use of NHPs to treat Osteoarthritis longer than 6 monthsN (%)**	21 (84)	9 (69)	12 (100)	0.096 ^**a**^
**Mean importance of scientific evidence rating (0 = unimportant, 10 = very important) (standard deviation)**	8 (2.3)	8 (2.6)	7.9 (2.1)	0.931^**c**^
**Mean overall health rating (0 = worst, 10 = best) (standard deviation)**	7.2 (1.2)	7.0 (1)	7.5 (1.4)	0.322 ^**c**^
**Mean pain scale visual analogue scale, 0 = worst, 10 = best (standard deviation)**	6.7 (1.8)	6.2 (2.0)	6.9 (1.6)	0.328^**c**^
**Joints affected**^**d**^**N (%)**				
**Upper extremity**	4 (21)	1 (10)	3 (33)	0.270^**b**^
**Lower extremity**	12 (63)	8 (80)	4 (44)	
**Other**	3 (16)	1 (10)	2 (22)	
**Total joints affected**	19 (100)	10 (100)	9 (100)	
**Recruitment location**^**b**^**– N (%)**				
**Senior community centre**	5 (20)	1 (20)	4 (80)	0.2505 ^**b**^
**Online community website**	17 (68)	11 (65)	6 (35)	
**Health food store**	2 (8)	1 (50)	1 (50)	
**Natural Health Products Used – N (%)**				
**Glucosamine**	25 (100)	13 (100)	12 (100)	
**Chondroitin**^**e**^	18 (72)	8 (62)	10 (83)	
**MSM**	0 (0)	0 (0)	10 (83)	
**Bromelain**	4 (16)	0 (0)	4 (33)	

Caption: a: Fisher test, b: Chi-square test, c: unpaired t-test; d: four participants did not indicate their income; e: chondroitin products were almost always in combination with glucosamine. All tests were performed using Prism GraphPad version 5.0 for Windows. Participants could indicate more than one anatomical joint; five participants did not indicate an affected anatomical joint.

When reading the HVMs in Figures 1 and 2, the numbers inside the boxes represent the percentage of all participants who mentioned the concept. The thickness of the lines connecting each of the concepts represents the strength of each linkage. The dominant chain that includes scientific evidence, described in the section below, captures the concepts used for product evaluation which relate to our central research question.

Participants in both the “high” and “low” scientific evidence groups described deciding to use NHPs because of perceptions about the scientific evidence for the efficacy or safety of the product, and endorsements by trusted individuals. Participants described being more likely to take NHPs when they expected fewer or no side effects. After taking the NHPs, participants reported experiencing reduction in pain, being able to perform more of their daily activities, and ultimately leading lives of improved quality and independence. An exploration of the salient 0product attributes, consequences, and values found in the dominant chain that includes scientific evidence is provided below.

#### Perceptions of scientific evidence

Participants expressed a general understanding that scientific evidence in our study was defined as *published research studies using the scientific method*. Both “high” and “low” participants endorsed scientific evidence as playing a role in their decision making. Scientific evidence was rated as important in their selection of NHPs (mean 8/10, scale 0= not important to10 = important) with no significant differences between the groups (See Table 1). Scientific evidence as a product attribute was discussed by 58% of the low scientific evidence group and 46% of those in the high scientific evidence group as a factor related to their product choice(s). Even though our participants appeared to understand the scientific method, and frequently linked the presence of scientific evidence with effectiveness such as a reduction in pain, there was no mention of *strength* of scientific evidence improving the *probability* of benefit. Instead, our participants frequently associated presence of scientific evidence with a decreased frequency of side effects.

During the semi-structured portion of the interview, most participants reported hearing about scientific evidence from an indirect source such as a friend or family member, a health care provider, media source or the internet; as opposed to directly seeking out scientific research studies to read. Scientific evidence was not described by most participants as a necessary condition for choosing a specific NHP to treat their OA. It was reported to be just “another nail in the coffin” and to be useful if it confirmed “what the other people said.” (High Evidence, M1).

Overall, our participants appeared to have a superficial understanding of scientific evidence. For example, none of them appeared to understand the significance of the evidence-based medicine’s (EBM’s) hierarchy of scientific evidence [28]. The EBM hierarchy is generally accepted in the research and clinical communities, and was adopted by the Natural Standard Database to determine levels of scientific evidence supporting NHPs.

#### Endorsements of trusted individuals

One of the most important product attributes influencing a participant’s selection of an NHP was the endorsement by someone the individual trusted. Friends and family were usually the initial, and often the most important, sources of information about NHP, including scientific evidence of efficacy:

Like I said, if my friend didn’t tell me that it worked, I wouldn’t know […] I didn’t do a search, no. I just took my friend’s word for it.

(High Evidence, M3).

If my son says take it, I’ll trust him. (Low Evidence, F5).

Endorsement of a product by a health care provider was also an important source of information. The types of health care providers consulted (CAM vs. conventional) did not significantly differ between the two groups of participants. Trust also appeared to be a key element in participants’ interactions with their health care providers:

I trust what he [my family doctor] says to me. I don’t think I’ve ever questioned anything he ever says to me. (High Evidence, M1).

Participants who discussed their physicians quoting scientific evidence usually recalled the information from their physicians as being negative or neutral about the efficacy of a NHP:

And [the doctor] said, well it’s not a product that really works. The doctor, he told me I could take a placebo pill, it would be the same way […] he didn’t say it was bad [or] don’t take it, it’s going to do negative things. He just said, it has no value. (Low Evidence, F2).

The advice of health care providers appeared to be playing slightly different roles in the decision making process for participants in the high and low scientific evidence groups. Participants in the high scientific evidence group related the endorsement of a health care provider to both the product’s efficacy (i.e. it decreased their pain) and how it worked (its mechanism of action). In contrast, participants in the low evidence category linked the knowledge that a product was endorsed by a health care provider with the perceived lack of (or fewer) side effects but did not identify a strong link to mechanism of action.

Participants in both scientific evidence categories talked about the NHP’s pain reduction ability as being key in allowing participants to perform their activities of daily living, feeling healthier physically, and emotionally, and being able to participate in social activities. Two key linkages mentioned by participants in the high scientific evidence category, but not mentioned by participants in the low scientific evidence category, were the importance of social activity and being in control of one’s health, both consequences which the participants linked with an improvement in quality of life.

Overall, two values (striving for an improved quality of life and maintaining independence) appear to be underpinning the decision to use NHPs for participants in both scientific evidence groups. Most participants in both groups described that experiencing less pain ultimately led to improved quality of life. “Quality of life” was defined by the participants as attaining more happiness, meaning, or purpose to live. Similarly, enabling improved activities of daily living led to greater levels of independence in both scientific evidence groups. In addition, more participants in the low scientific evidence group tended to associate the natural source of NHPs with being able to adopt a “natural way of living.” Only one participant in the low scientific evidence group made such a link (which means it is not present in the HVM because it was below the cut-off of three).

## Discussion

According to the Deficit Model participants who were in the high scientific group (i.e., who only used products supported by scientific evidence) should have reported more exposure to, and place more value on, scientific evidence than those purchasing products without scientific evidence of efficacy. This was *not* what we found. Participants in both groups appeared to have a very general understanding of the scientific method and identified scientific evidence as a factor in their decision-making. Upon detailed questioning in the semi-structured interviews, scientific evidence was not of primary importance to participants in either group. Further, participants who cited scientific evidence as a product attribute frequently associated the scientific evidence supporting an NHP with a reduction in side effects instead of acknowledging the greater probability of benefit of NHPs with higher levels of scientific evidence according to the EBM hierarchies of evidence [28]. This suggests that a reduction in side effects could be more personally meaningfully to many CAM users, regardless of probability of efficacy, a finding reported in previous CAM decision-making studies [11,13,29].

We believe that the Contextual model is helpful at explaining our findings since it recognizes that it is not simply an understanding of scientific facts that it is important when people make decisions, but rather how that information fits into their everyday experiences, concerns and values [17]. The Contextual Model can also explain the role of trust in individuals with authority (such as health care providers), as well as the profound influence of endorsements from friends and family to an individual’s decision-making process.

Our participants identified scientific evidence as a product attribute impacting their selection of NHPs, which is consistent with previous qualitative study findings where patients with arthritis [12], breast cancer [13-15], and prostate cancer [30,31] reported using scientific evidence as an information source guiding their decision making process when selecting CAM treatment therapies. While participants in this study identified scientific evidence as important, more detailed interviewing revealed that scientific evidence played a relatively unimportant role in decision-making compared to other factors such as personal advice. The reason our findings appear to differ from previous CAM decision-making studies could be our in-depth semi-structured interview questions which probed participants’ views of scientific evidence and allowed us to directly explore the relative importance of scientific evidence compared to other factors.

Similar to other CAM decision-making studies in patients diagnosed with cancer, most of our participants did not report going to the scientific research to read it first-hand, instead, they considered indirect sources such as internet searches, newspaper-articles, or health care providers’ endorsements sources of scientific evidence [14,15,32,33]. Proponents of the Contextual model suggest that individuals will choose indirect forms of scientific evidence because they have more personal relevance [16], and not because there is a lack of access to direct scientific evidence. This is something which could be explored more in future studies.

The MEC approach is based on an assumption that product selection is ultimately driven by attempts to fulfill an end or core value. Three core values emerged in our findings – desiring a natural way of living, quality of life, and independence. The most distinct value which appears to characterize participants who selected products without scientific evidence support was the desire for a “natural way of living”. This is consistent with previous research which suggests that individuals who use CAM appear to value a holistic way of living and the connection between the mind and body [12,13,15], not shared by those who only use conventional medicine [12]. A preference for things natural has also been found to be important in food choices [34-37].

A previous study by our team found that in consumers selecting sleep aids, individuals’ product selection process included making different tradeoffs between product attributes (e.g., naturalness) and perceived consequences (e.g., efficacy and side effects). These tradeoffs were done in an effort to maximize values such as overall quality of life [11]. Future research should focus on how and when these tradeoffs are made by consumers as well as how positioning consumer communication in personally meaningful contexts impacts them.

There are some limitations to this study. For example, we had a relatively small sample size (n =25). Nonetheless our sample (over 75% female participants, with a mean age of over 58 years, of North American heritage, consisting of fairly well educated (>60% with college education) individuals with over half earning more than $50,000) has similar demographics to the target group -- individuals with arthritis who choose to use NHPs or CAM therapies in general [7,12,38]. This suggests that our results capture the decision-making process of our target population. The participants who used NHPs with low levels of scientific evidence support could have also used NHPs with high levels of scientific evidence support, which could explain why there were relatively few differences between the two groups. However, our decision to allocate participants into groups based on their revealed preferences (i.e., based on the NHPs they actually used), rather than based on their stated preferences, enhances the validity of our findings. Most of our participants stated that scientific evidence of efficacy was important in their decision-making; yet their product choices suggest this is not always the case. Lastly, we did not test for scientific literacy; however, there was no significant difference between the education levels of both groups which is a proxy for scientific literacy.

## Conclusion

In conclusion, this study found that scientific evidence does play a role in consumer decision-making about NHPs; however, it is less important than the advice provided by health care providers, friends and family. Very few participants used direct scientific evidence sources in the decision-making process; most acquired knowledge of scientific evidence through indirect sources such as health care providers or the media, highlighting the responsibility of these groups to critically assess the quality and validity of scientific evidence since consumers/patients rely on them for accurate information. The Contextual Model of the Public Understanding of Science helps to explain why scientific evidence is not the most important decision-making factor because it helps us understand how perceived personal relevance is used to filter all information relevant to these types of decisions. Future research should focus on the content of the interactions between patients and heath care providers regarding scientific evidence, the perceived importance of different indirect sources of scientific evidence, and knowledge translation strategies to more effectively convey scientific evidence to the lay audience in ways they find personally meaningful.

## End notes

^1.^ Products and therapies generally defined as not being part of the dominant health care system.

^2.^ Evidence level A or B only. Level A evidence is strong scientific evidence, where level B is good scientific evidence, both according to the Natural Standard Database.

^3.^ Evidence level C or traditional/insufficient evidence. Level C evidence is unclear or conflicting scientific evidence according to the Natural Standard Database.

## Abbreviations

CAM: Complementary and alternative medicine; EBM: Evidence-based medicine; HVM: Hierarchical value map; MEC: Means-end chain; MSM: Methylsulfonylmethane; NHP: Natural health product; OA: Osteoarthritis.

## Competing interests

The authors have no competing interests.

## Authors’ contributions

TT and HB conceived of the study and its design. TT and NK conducted the interviews. HB, TT, and NK participated in the analysis of the means end chain data and semi-structured interviews. AB and MK participated in refining the study design, in the means-end chain data-analysis and interpretation of the data. TT drafted the manuscript. All authors reviewed and revised the manuscript and approved the final version.

## Authors’ information

TT, MSc ND

Research Network Coordinator, Canadian Interdisciplinary Network for CAM Research (IN-CAM), Leslie Dan Faculty of Pharmacy, University of Toronto, Ontario, Canada.

Clinic Supervisor and Academic Instructor, Canadian College of Naturopathic Medicine, Toronto, Ontario, Canada.

HB, BScPhm, PhD

Professor and Associate Dean, Graduate Education, Leslie Dan Faculty of Pharmacy, University of Toronto, Toronto, Ontario, Canada.

AB, PhD

Associate Professor and Undergraduate Coordinator, Department of Food, Agricultural & Resource Economics, FARE, University of Guelph, Guelph, Ontario, Canada.

NK, MEd

Leslie Dan Faculty of Pharmacy, University of Toronto, Ontario, Canada

MK, MD MSc, FRCPC

Director of THETA, the F. Norman Hughes Chair in Pharmacoeconomics, Leslie Dan Faculty of Pharmacy, University of Toronto, Toronto, Ontario, Canada.

Professor in the Faculty of Medicine and Leslie Dan Faculty of Pharmacy, University of Toronto, Toronto, Ontario, Canada.

Scientist Toronto General Research Institute, Toronto, Ontario, Canada.

Adjunct Scientist, Institute for Clinical Evaluative Sciences, Toronto, Ontario, Canada.

## Pre-publication history

The pre-publication history for this paper can be accessed here:

http://www.biomedcentral.com/1472-6882/12/198/prepub
